# Short-term effects of air pollution on blood pressure

**DOI:** 10.1038/s41598-019-56413-y

**Published:** 2019-12-30

**Authors:** You-Jung Choi, Sun-Hwa Kim, Si-Hyuck Kang, Sun-Young Kim, Ok-Jin Kim, Chang-Hwan Yoon, Hae-Young Lee, Tae-Jin Youn, In-Ho Chae, Cheol-Ho Kim

**Affiliations:** 1 Cardiovascular Center, Seoul National University Bundang Hospital, Seongnam-si, Korea; 2 Department of Internal Medicine, Seoul National University, Seoul, Korea; 3 Department of Cancer Control and Population Health, Graduate School of Cancer Science and Policy, National Cancer Center, Goyang-si, Gyeonggi-do Korea; 4 Cardiovascular Center, Seoul National University Hospital, Seoul, Korea

**Keywords:** Environmental impact, Renovascular hypertension

## Abstract

Elevated blood pressure (BP) has been proposed as a possible pathophysiological mechanism linking exposure to ambient air pollution and the increased risk of cardiovascular mortality and morbidity. In this study, we investigated the hourly relationship between ambient air pollutants and BP. BP measurements were extracted from the electronic health record database of the Seoul National University Bundang Hospital from February 2015 to June 2017. A total of 98,577 individual BP measurements were matched to the hourly levels of air pollutants. A generalized additive model was constructed for hour lags of 0–8 of air pollutants adjusting for age, sex, meteorological variables, and time trend. Systolic BP was shown to be significantly lower at 2–4 hours and 3–5 hours after increased levels of SO_2_ and CO, respectively (0.24 mmHg and 0.26 mmHg for an interquartile range, respectively). In contrast, O_3_ and NO_2_ were associated with significantly increased systolic BP at 3–5 lag hours and at 0–2 lag hours, respectively. BP elevation in association with O_3_ and NO_2_ was shown to be significantly greater in hypertensive patients than normotensive subjects. Our findings suggest that short-term exposure to air pollution may be associated with elevated BP.

## Introduction

Elevated blood pressure (BP) is the top leading risk factor for morbidity and mortality in both men and women^1^. Every 20 mmHg increase in systolic BP (or 10 mmHg increase in diastolic BP) has been shown to be associated with more than a two-fold increase in cardiovascular mortality^2,3^. It is currently estimated that over 30% of adults have hypertension (defined as systolic and/or diastolic BP ≥140/90 mmHg) worldwide^4^. BP is variable and can be affected by various environmental factors. Behavioral factors, including high dietary salt intake, obesity, physical inactivity, and alcohol abuse contribute to elevated BP^5–7^. Effects of environmental factors such as temporal trends, ambient temperature, and noise are also well known^8^. Elevated short-term BP variability and pathologic diurnal patterns have been shown to be associated with poor prognosis^9,10^.

Ambient air pollution is a major cause of death and disease. The World Health Organization (WHO) estimates that 7.6% of total global deaths are attributable to ambient air pollution^11^. The Global Burden of Disease study ranked ambient air pollution as sixth among other risk factors in terms of attributable disability-adjusted life-year^12^. Most of the disease burden associated with ambient fine particulate matter (PM) (PM with an aerodynamic diameter of <2.5 μm, PM_2.5_) is attributed to cardiovascular disease including ischemic heart disease and cerebrovascular disease. The estimated burden is particularly high in Asian countries such as China and India.

Recent studies have suggested that air pollution leads to elevated BP, and this may be an important mechanism defining the link between ambient air pollution and cardiovascular mortality and morbidity^13,14^. Studies have demonstrated a rapid increase in diastolic BP after controlled exposure to PM_2.5_ and ozone (O_3_)^15,16^. Observational studies and meta-analyses have also indicated that short-term exposure to air pollution, including PM_2.5,_ increases BP and the frequency of emergency visits for hypertension^17–19^. In addition, long-term exposure to PM_2.5_ and nitrogen dioxide (NO_2_) has been associated with higher BP and development of hypertension^19,20^.

Most previous short-term effect studies utilizing a population-based database had limitations of assessing binary outcomes such as emergency visits for hypertension rather than absolute BP values^18,21,22^. In addition, because of constraints in time resolution, analyses were mostly limited to day lag models but reports including hour lag models have been scarce. Several studies that assessed hourly changes in BP comprised small sample sizes, which made it difficult to fully appreciate the association with sufficient statistical power^15,16,23^. Therefore, this study sought to investigate the short-term relationship between ambient air pollution and BP using electronic health record data from patients visiting a tertiary center in Korea. We hypothesized that air pollution contributes to pathological short-term BP changes, which may, in turn, lead to adverse cardiovascular outcomes. Large volumes of electronic health record data combined with a high temporal resolution enabled the examination of short-term effects of air pollution on BP on an hourly basis.

## Results

### Study subject profiles, air pollution levels, and meteorological data

A total of 98,577 subjects with available BP measurements were analyzed in this study. As shown in Table 1, mean age was 55.5 years and 50.2% were male. Among the study subjects, 31.7% had hypertension, 2.2% diabetes, and 10.4% dyslipidemia. The mean systolic and diastolic BP was 122.8 ± 16.3 mmHg and 74.1 ± 11.1 mmHg, respectively. Mean PR was 79.3 ± 13.4 per minute. Most measurements (97.8%) were obtained between 9 a.m. to 6 p.m. (Supplementary Fig. 1). Subjects with hypertension were older and more frequently associated with male sex and comorbidities. Calcium channel blockers were the most commonly used antihypertensive medication.

**Table 1:** Descriptive statistics on study subjects and blood pressure measurements.

Values	Total (n = 98,577)	Hypertension (n = 31,267)	Normotension (n = 67,310)
Age, years	55.5 ± 14.9	61.8 ± 14.1	52.6 ± 14.4
Male sex, n (%)	49,452 (50.2)	16,866 (53.9)	32,586 (48.4)
Body mass index, kg/m^2^*	23.83 ± 0.49	25.16 ± 0.41	23.83 ± 0.49
Blood pressure measurements			
Systolic blood pressure, mmHg	122.8 ± 16.3	127.1 ± 17.8	120.8 ± 15.1
Diastolic blood pressure, mmHg	74.1 ± 11.1	75.6 ± 11.8	73.4 ± 10.6
Pulse rate, per minute	79.3 ± 13.4	78.3 ± 13.8	79.8 ± 13.2
Comorbidities			
Diabetes mellitus, n (%)	7553 (7.7)	5732 (18.3)	1821 (2.7)
Dyslipidemia, n (%)	10,288 (10.4)	6584 (21.1)	3704 (5.5)
**Medication, n (%)**			
Antihypertensive agents			
ACEI/ARB	13,582 (13.8)	13,582 (43.4)	—
Calcium channel blockers	17,505 (17.8)	17,505 (56.0)	—
β-blockers	17,090 (17.3)	17,090 (54.7)	—
Diuretics	7565 (7.7)	7565 (24.2)	—
Statin	17,813 (18.1)	13,632 (43.6)	4181 (6.2)

Data are reported as numbers and percentages for categorical variables and mean ± standard deviation (SD) for continuous ones. *Body mass index was only available in 59,215 subjects (61.1%).

ACEI denotes angiotensin-converting-enzyme inhibitor; ARB, angiotensin II receptor blockers.

Table 2 summarizes the meteorological and air pollution data obtained during the study period. The mean concentrations of PM_2.5_, O_3_, and NO_2_ were 23 μg/m^3^, 17 ppb, and 29 ppb, respectively. The mean temperature was 13.4 °C. Supplementary Table 1 shows the correlation matrix between air pollutants, temperature, and relative humidity during the study period. PM_2.5_, PM_10_, SO_2_, CO showed moderate relationships with each other. NO_2_ and O_3_ showed more dramatic diurnal variations: O_3_ level was highest during daytime when NO_2_ concentration was lowest (Supplementary Fig. 2).

**Table 2:** Descriptive statistics on environmental variables during the study period.

Values	Total
**Air pollutants, median (interquartile range)**	
PM_2.5_, μg/m^3^	23 (18)
PM_10_, μg/m^3^	44 (33)
SO_2_, ppb	4 (2)
CO, ppm	0.5 (0.2)
O_3_, ppb	17 (26)
NO_2_, ppb	29 (24)
**Meteorological data, mean (SD)**	
Temperature, °C	13.4 (18.2)
Atmospheric pressure, hPa	1017.0 (13.3)
Humidity, %	66 (37)

Medians (IQR) or mean (standard deviation) are presented.

PM_2.5_ denotes fine particulate matter (<2.5 µm); PM_10_, fine particulate matter (<10 µm); SO_2_, Sulfur dioxide; CO, carbon monoxide; O_3_, ozone; NO_2_, Nitrogen dioxide.

### Short-term relationship between air pollutants and blood pressure

**Figure 1:**
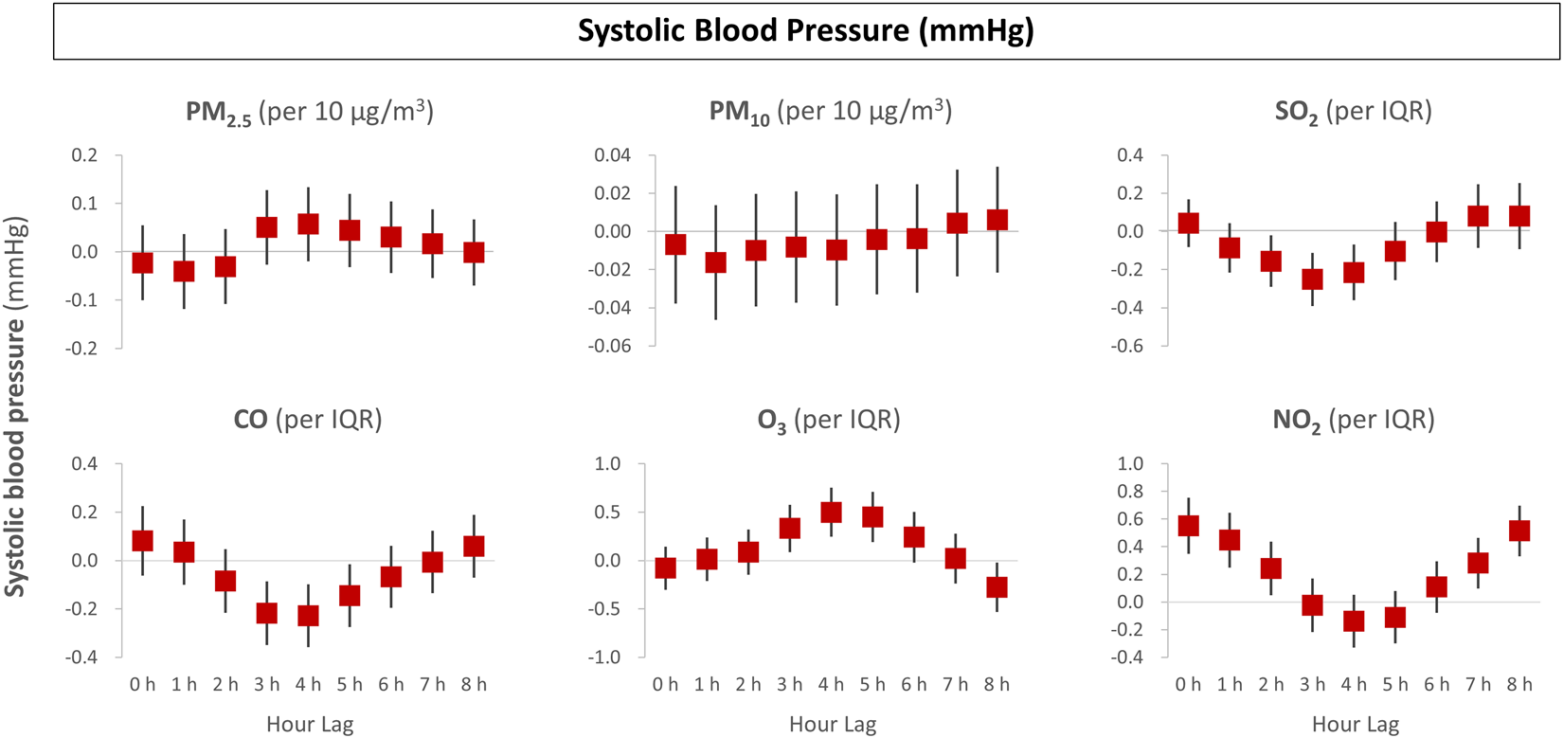
Time-lag effects of air pollution on systolic blood pressure. The x-axis represents hour lags, while the y-axis indicates adjusted effects on systolic blood pressure.

Different pollutants showed different effects with varying time lags. Systolic BP had no significant association with particulate matter of PM_2.5_ or PM_10_ from 0 to 8 hours before BP measurements. Nonetheless, gaseous pollutants showed significant lag effects with systolic BP (Fig. 1). SO_2_ and CO significantly reduced systolic BP at 2–4 lag hours and at 3–5 lag hours, respectively, while O_3_ and NO_2_ were associated with elevated systolic BP at 3–5 lag hours and at 0–2 lag hours, respectively.

**Figure 2:**
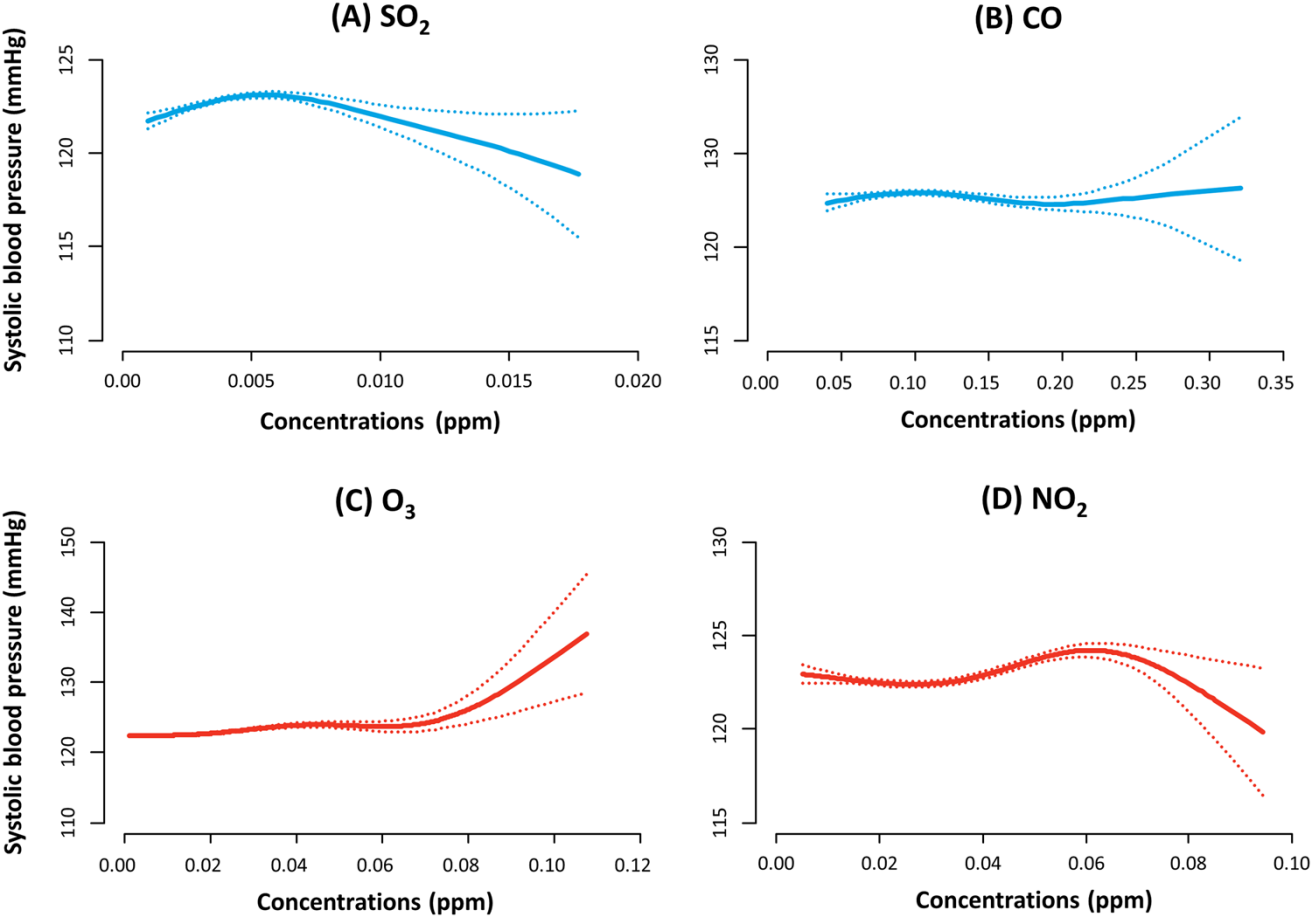
Spline curves showing the non-linear relationship of (**A**) SO_2_, (**B**) CO, (**C**) O_3_, and (**D**) NO_2_ with systolic blood pressure. The running mean of lag hours 3–5 was used for SO_2_ and O_3_, and that of lag hour 2–4 for CO and lag hours 0–2 for NO_2_.

Figure 2 shows the nonlinear relationship of running means of SO_2_ (3–5 lag hours), CO (2–4 lag hours), O_3_ (3–5 lag hours) and NO_2_ (0–2 lag hours) with systolic BP. An IQR increase of O_3_ (26 ppb) was associated with an increase in systolic BP by 0.89 ± 0.10 mmHg at 3–5 lag hours when unadjusted and by 0.55 ± 0.14 mmHg when adjusted. An IQR increase of NO_2_ (24 ppb) was associated with an increase in systolic BP by 0.65 ± 0.09 mmHg at 0–2 lag hours when unadjusted, and by 0.42 ± 0.12 mmHg after multivariable adjustment. In the meantime, SO_2_ and CO were shown to be associated with increased systolic BP: −0.24 ± 0.08 mmHg at 2–4 lag hours by an IQR of SO_2_ (2 ppb) and −0.26 ± 0.07 mmHg at 3–5 hour lags by an IQR of CO (0.2 ppm)

Time lag effects of air pollutants on diastolic BP were generally consistent with those of systolic BP (Supplementary Fig. 3). SO_2_ and CO significantly reduced diastolic BP, while O_3_ and NO_2_ were associated with elevated diastolic BP. None of the pollutants showed significant associations with PR (Supplementary Fig. 4).

### Subgroup analysis

**Figure 3:**
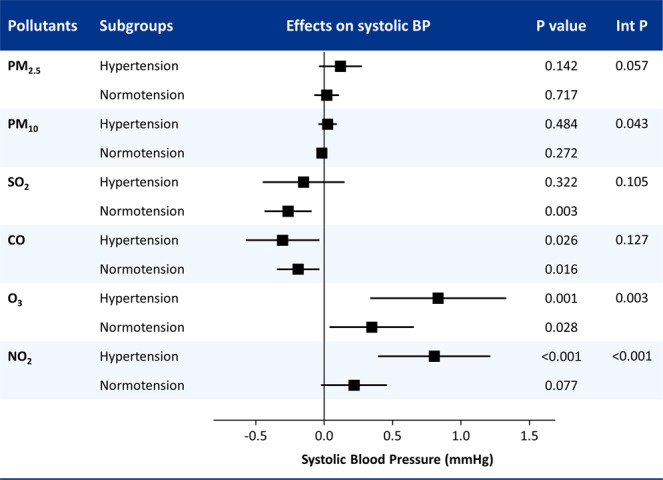
Subgroup analysis for the associations between air pollutants and systolic blood pressure (BP) stratified by the presence of hypertension. Int P, interaction P values; PM2.5, fine particulate matter with an aerodynamic diameter of <2.5 µm; PM10; CO, carbon monoxide; SO_2_, sulfur dioxide; NO_2_, nitrogen dioxide; O_3_, ozone.

Effect estimates of air pollutants on BP were analyzed stratified by the presence of hypertension (Fig. 3). The relationship of O_3_ and NO_2_ with systolic BP showed significant interaction with hypertension. BP-elevating effects of O_3_ and NO_2_ were significantly greater in patients with hypertension than in normotensive subjects. Although there was a significant interaction, the impact of PM_10_ was not significant in either of the subgroups. PM_2.5_ showed a similar trend without statistical significance. No significant interaction was present for SO_2_ and CO levels. No significant interactions were observed between BP and BMI across air pollutants (Supplementary Fig. 5).

## Discussion

The present study evaluated the short-term relationship of ambient air pollution and BP. BP was shown to be elevated within several hours after exposure to increased levels of O_3_ and NO_2_, and to be depressed after exposure to SO_2_ and CO. PM showed no significant short-term effects on BP. The increase in systolic BP related with short-term exposure to O_3_ and NO_2_ was significantly greater in hypertensive than in normotensive subjects.

Air pollution contributes to cardiovascular mortality and morbidity^24–27^. Proposed pathways include increased systemic inflammation, oxidative stress, endothelial dysfunction, atherogenesis, altered autonomic control, arrhythmogenesis, and an increase in BP^13,14^. Studies linking air pollution and arterial BP have shown conflicting results. Several epidemiologic studies have suggested acute increases in systemic BP with higher daily PM levels (effect magnitudes ranging from 1- to 4-mmHg increase in systolic and diastolic BP per 10 µg/m^3^ elevation in PM)^28–31^. However, other studies failed to show any significant associations^32–34^. Several distinctive features make the present study different from the previous ones. First, the time frame considered was hours after exposure, which is much shorter than previous short-term studies that were mostly based on day lag model analysis. Second, while previous studies have provided strong evidence for adverse cardiovascular effects of PM, the present study showed that extremely short-term effects on BP were greater with gaseous pollutants.

Ozone causes oxidative stress. Studies have shown there is a short-term increase in cardiovascular mortality in response to O_3_ exposure^35,36^. A recent quasi-experimental study also showed that short-term O_3_ exposure leads to an increase in BP in healthy adults^37^. A possible mechanism linking elevated BP and O_3_ exposure is increased serotonin-induced vasoconstriction and decreased acetylcholine-induced vasodilation^38^. NO_2_ is produced from the combustion of fossil fuel in industrial factories or automobiles. Previous studies have yielded conflicting results regarding the short-term effects of NO_2_ on BP^19,39^. This inconsistency between previous studies might be partly explained by the difference in study methodology such as exposure time and study sample size, as well as population susceptibility and regional differences.

The present study showed negative relationships of CO and SO_2_ with systolic BP. CO is the product of incomplete combustion of carbon. Vascular effects of CO have been reported to resemble those of nitric oxide (NO). Experimental studies have suggested endogenously produced CO contributes to vasorelaxation in the cerebrovascular circulation^40^. Penney and his collaborators have performed thorough animal studies to find that the response to CO consists of systemic arterial hypotension and reduced systemic vascular resistance^41,42^. A human study also showed decreased peripheral resistance after acute exposure to CO^43^.

The burning of fossil fuels by power plants and other industrial facilities accounts for the largest source of SO_2_ in the atmosphere. Experimental studies demonstrated acute exposure to SO_2_ and SO_2_ derivatives (SO_x_) had a dose- and time-response relationship with a decrease in BP in rat models^44^. Vasodilatation is suggested as the key mechanism that SO_2_ inhibits Ca^2+^ entry through both potential-dependent calcium channels and receptor-operating calcium channels, and intracellular Ca^2+^ release^45^.

In the present study, we found no significant short-term relationship between PM and BP. Evidence supporting longer-term effects of PM air pollutants on hypertension is more convincing. Studies have linked short- and long-term exposure to PM and the risk of hypertension^19,46^. In addition, a randomized trial showed an intervention to reduce indoor air pollution resulted in improved BP control^47^. PM causes systemic inflammatory/oxidative response and autonomic dysfunction, which may in turn leads to elevated BP^13^. Therefore, long lags (than within several hours) may be required to observe meaningful response in BP.

This study has several limitations that originate from the study design. First, the changes in systolic BP was no greater than 1 mmHg per IQR, which confers little clinical significance. Second, this study was observational and provides little insight into the pathophysiology. Although different pollutants showed significant associations at different time points in this study, future studies are required to understand the differential effects. Third, concentrations measured in a nearby monitoring station were used as a proxy for an individual’s exposure to pollutants, which is a potential source of bias. However, measuring an individual’s exposure to air pollutants requires special efforts including portable devices, which is difficult to achieve for large-scale studies such as the present study. This study was one of the largest to measure absolute BP levels and analyze short-term effects of air pollution. Fourth, a further limitation is the representativeness of the study sample. The present study participants were limited to patients of a tertiary medical center. Fifth, BP was not measured in a controlled condition. Recent studies have suggested the importance of ‘white-coat effects’, which means measured BP values may vary widely according to the measurement methods^48^. Sixth, this study evaluated between-subject differences in BP only. Because the number of repeated measurements varied according to the subjects, we were not able to analyze within-subject BP changes. Finally, while this study aimed at evaluating very short-term relationship, we could not assess day lag effects with this study design.

In summary, this study suggests that air pollution affects BP within hours after exposure. Gaseous pollutants such as O_3_ and NO_2_ increased systolic and diastolic BP in the short term. Patients with hypertension were shown to be more vulnerable to the BP-elevating effects than normotensive subjects. Short-term effects of PM were not significant, while SO_2_ and CO were associated with a reduction in BP within hours.

## Methods

### Study database and study participants

A retrospective analysis of single-center electronic health record data was performed. The study individuals were extracted from the Seoul National University Bundang Hospital clinical data warehouse. The center is a tertiary referral hospital in Korea and is equipped with complete electronic medical records reaching stage 7 criteria according to the Electronic Medical Record Adoption Model of the Healthcare Information and Management System Society. This study conformed to the principles of the Helsinki declaration of 1975 (revised version 2013). The study protocol was approved by the institutional review board of Seoul National University Bundang Hospital (No. B-1709-420-003), which waived the need for written informed consent from the subjects due to the retrospective nature of the study.

All consecutive subjects aged 18 years or older who were ambulatory and visited outpatient clinics with available BP measurements from February 2015 to June 2017 were enrolled. Subjects who visited the emergency department were excluded. The levels of systolic and diastolic BP, and pulse rate (PR) as well as the exact time of measurements (date, hour, minutes, and seconds) were extracted. Information on demographics, comorbidities, and medication history was collected from the data warehouse. Hypertension was defined as International Classification of Diseases, 10th Revision (ICD-10) code I10‒I15 and/or taking antihypertensive medications; diabetes as ICD-10 code (E11‒14) and/or glucose-lowering agents; and dyslipidemia as ICD-10 code (E78) and/or the use of lipid-lowering therapy.

### Blood pressure measurement

BP was measured using automated oscillometric devices, including HBP-9020 (Omron Healthcare Co., Ltd., Japan), TM-2655 (Kensei Industry Co., Ltd., Japan), and Easy-X 800 R (Jawon Medical Co. Ltd, Republic of Korea), which were equipped with an elbow detection sensor and movable cuff to provide accurate measurements. Upon visiting the outpatient department, patients were instructed to measure their own sitting BP after resting for over 5 minutes. Two or more measurements were generally recommended, but not mandatory. Measured values were automatically matched to the patients’ identification code and recorded in the electronic health record database.

When a subject performed multiple measurements of BP within an hour, the mean of the last two values was chosen. Two measurements more than an hour apart were considered separate measurements. If a subject made multiple visits during the study period, the first value was chosen because only between-subject effects and not within-subject effects were considered in this study. The primary study endpoint was systolic BP, while secondary endpoints included diastolic BP and PR. The rationale for systolic BP as the primary endpoint is the recent evidence showing that systolic BP has greater effect on adverse outcomes than diastolic BP ^3,49^.

### Air pollution and meteorological data

Hourly average concentrations of PM_2.5_, PM with an aerodynamic diameter of <10 μm (PM_10_), carbon monoxide (CO), sulfur dioxide (SO_2_), NO_2_, and O_3_ were obtained from the nearest monitoring station of the air quality regulatory monitoring network. The station (Unjung-Dong station) is located 5.9 km from the hospital and is installed in a residential area with a purpose to monitor urban air quality. This station collects air samples from 10‒20 meters above the ground level and indicates air quality within 70 km. PM_2.5_ and PM_10_ were measured by the β-ray absorption method, CO by the non-dispersive infrared method, SO_2_ by pulse ultraviolet fluorescence, NO_2_ by chemiluminescence, and O_3_ by ultraviolet photometry. Meteorological data, including temperature (°C), relative humidity (%), and air pressure (hPa) were obtained from the nearest station 15.1 km from the meteorological observation network run by the Korean Meteorological Administration.

### Statistical analyses

Each BP and PR measurement was matched to the hourly levels of air pollutants and meteorological variables by linking the date and the time of measurements. Hourly BP or PR, hourly air pollution, smoothed meteorology, and an hourly time trend were put into generalized additive model with a Gaussian link function. Adjusted variables included study subjects’ demographic factors (age and sex), meteorological variables (temperature, atmospheric pressure, and humidity), time variables (year, month, day of the week, and hourly time trend). Natural splines were used for smoothing independent variables. Three degrees of freedom were chosen for temperature, atmospheric pressure, and humidity, while 8 for an hourly time trend based on explorations using 1 to 10 degrees of freedom. To investigate delayed effects of air pollution on BP or PR, we estimated the effects of air pollution on the concurrent hour through 8 hours prior to BP or PR. Eight-hour lag was chosen as the maximum lag because outpatients rarely stay more than 8 hours (working hour: 9 a.m.‒5 p.m.). A nonlinear relationship between air pollution levels and systolic BP was illustrated with the smooth function using penalized splines. Subgroup analysis was performed for the presence of hypertension and body mass index (BMI) (<25 kg/m^2^ and ≥25 kg/m^2^). Running means of lag hours during which the association was significant were used as exposure (lag hour 3 to 4 for PM_2.5_, 2 to 4 for SO_2_, 3 to 5 for CO, 3 to 5 for O_3_, and 0 to 2 for NO_2_). Descriptive statistics on study population were reported as means ± standard deviation (SD) for continuous variables and as numbers (percentages) for categorical variables. Effect estimates of BP or PR were presented for interquartile increments, as the difference between the 25th and 75th percentiles, for the concentration of each air pollutant.

A two-sided p-value < 0.05 was considered statistically significant. All statistical analyses were performed using R programming version 3.2.4 (http://www.R-project.org; The R Foundation for Statistical Computing, Vienna, Austria).

## Supplementary information

Supplementary material
