# Supplementary material for: Short-term effects of air pollution on blood pressure
Source: Sci Rep. 2019 Dec 30;9:20298. doi: 10.1038/s41598-019-56413-y (PMC6937254; doi:10.1038/s41598-019-56413-y)
Supplement: Supplementary file 1 — Supplementary material [file 41598_2019_56413_MOESM1_ESM.pdf]

Short-term effects of air pollution on blood pressure

Short title: Short-term effects of air pollution on BP

You-Jung Choi, MD[a,b,d], Sun-Hwa Kim, PhD[a], Si-Hyuck Kang, MD[a,b], Sun-Young Kim, PhD[c], Ok-Jin Kim, PhD[c], Chang-Hwan Yoon, MD, PhD[a,b], Hae-Young Lee, MD, PhD[b,d], Tae-Jin Youn, MD, PhD[a,b], In-Ho Chae, MD, PhD[a,b], Cheol-Ho Kim, MD, PhD[a,b]

[a]Cardiovascular Center, Seoul National University Bundang Hospital, Seongnam-si, Korea;

[b]Department of Internal Medicine, Seoul National University, Seoul, Korea;

[c]Department of Cancer Control and Population Health, Graduate School of Cancer Science and Policy, National Cancer Center, Goyang-si, Gyeonggi-do, Korea;

[d]Cardiovascular Center, Seoul National University Hospital, Seoul, Korea

Address for correspondence:

Si-Hyuck Kang, MD

Cardiovascular Center, Internal Medicine, Seoul National University Bundang Hospital

82, Gumi-Ro 173 Beon-Gil, Bundang-Gu, Seongnam-Si, Gyeonggi-Do, Korea, 13620

Telephone: 82-31-787-7027; Fax: 82-31-787-4290; E-mail: eandp303@snu.ac.kr

Supplementary Material

Supplementary Figure 1. Histogram of the hour a day when blood pressure and pulse rate measurements were done

Supplementary Table 1. Correlation matrix among air pollutants, temperature, and humidity during the study period

Supplementary Figure 2. Relative concentrations of air pollutants according to time in a day during the study period

Supplementary Figure 3. Time-lag effects of air pollution on diastolic blood pressure

Supplementary Figure 4. Time-lag effects of air pollution on pulse rate

Supplementary Figure 5. Subgroup analysis for the associations between air pollutants and systolic blood pressure stratified by body mass index

Supplementary Figure 1. Histogram of the hour a day when blood pressure and pulse rate measurements were done

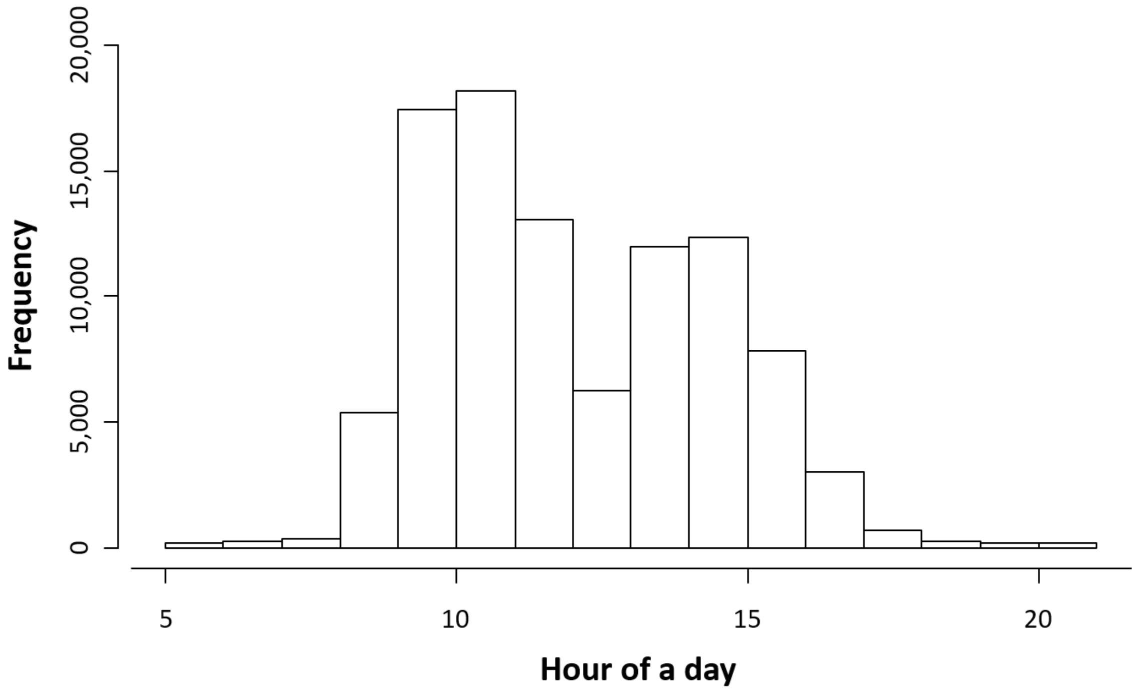

Supplementary Table 1. Correlation matrix among air pollutants, temperature, and humidity during the study period

	$PM_{2.5}$	PM_{10}	SO_2	CO	O_3	NO_2	Temperature	Humidity
**$PM_{2.5}$**	–	0.64	0.40	0.58	0.09	0.33	-0.05	0.07
**PM_{10}**		–	0.34	0.42	0.07	0.21	-0.16	-0.10
**SO_2**			–	0.49	0.05	0.33	-0.20	-0.30
**CO**				–	-0.37	0.67	-0.37	0.17
**O_3**					–	-0.58	0.50	-0.40
**NO_2**						–	-0.31	0.13
**Temperature**							–	0.03
**Humidity**								–

Abbreviations: $PM_{2.5}$, fine particulate matter with an aerodynamic diameter of <2.5 µm; PM_{10}, fine particulate matter with an aerodynamic diameter of <10 µm; CO, carbon monoxide; SO_2, sulfur dioxide; NO_2, nitrogen dioxide; O_3, ozone.

Supplementary Figure 2. Relative concentrations of air pollutants according to time in a day during the study period

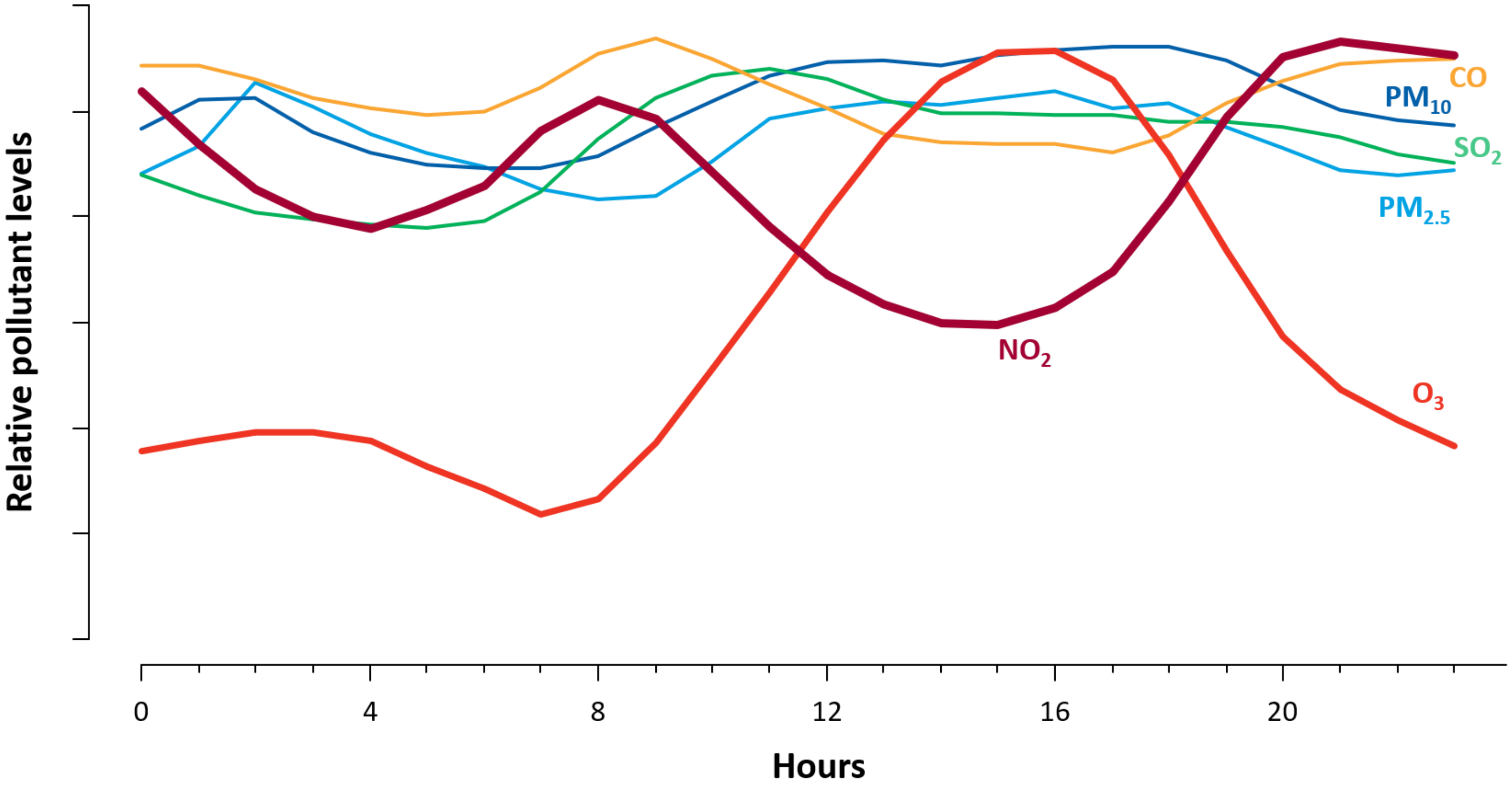

Abbreviations: $PM_{2.5}$, fine particulate matter with an aerodynamic diameter of <2.5 µm; PM_{10}, fine particulate matter with an aerodynamic diameter of <10 µm; CO, carbon monoxide; SO_2, sulfur dioxide; NO_2, nitrogen dioxide; O_3, ozone.

Supplementary Figure 3. Time-lag effects of air pollution on diastolic blood pressure

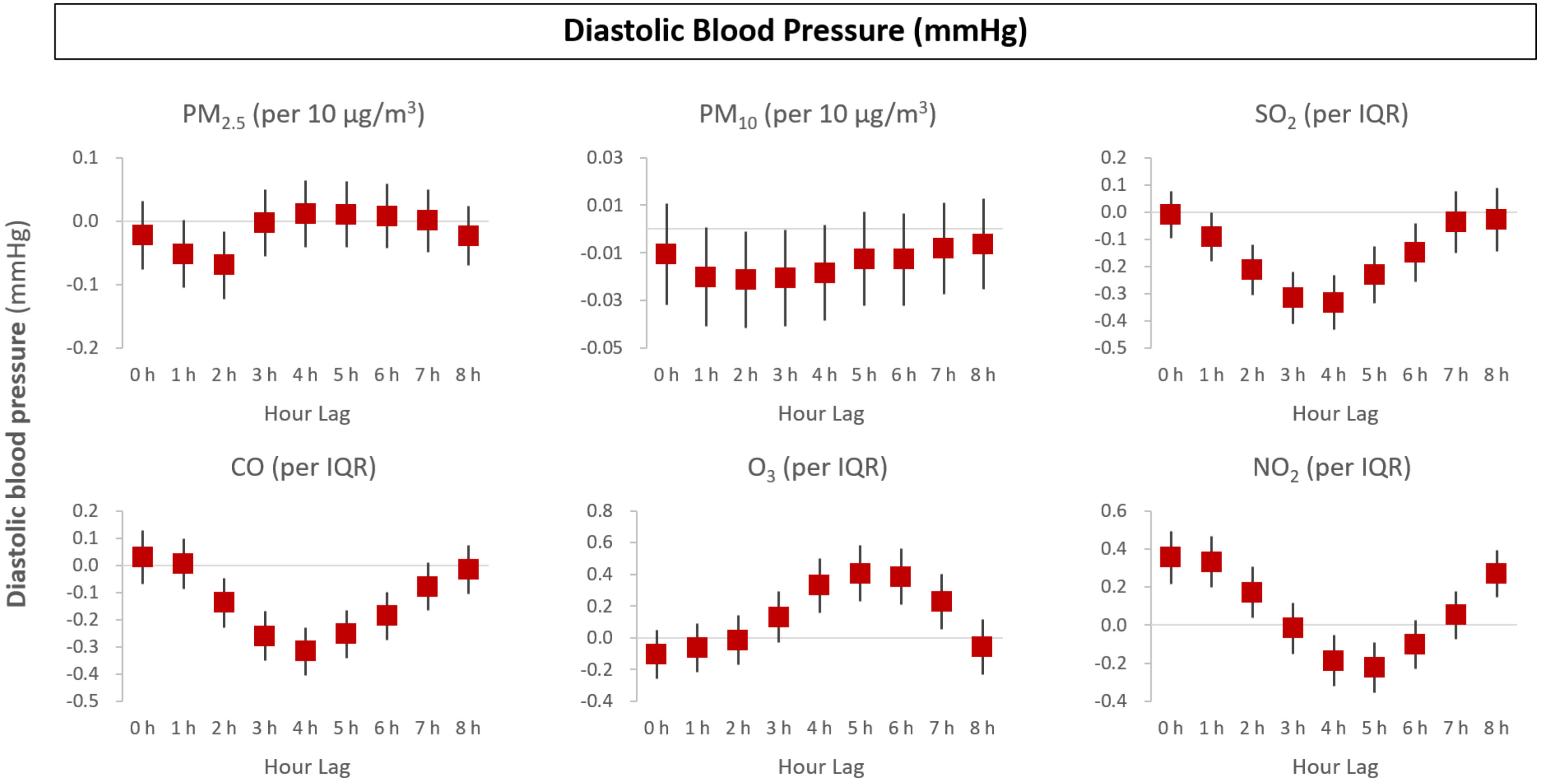

The x-axis represents hour lags, while the y-axis does adjusted effects on systolic blood pressure.

Abbreviations: $PM_{2.5}$, fine particulate matter with an aerodynamic diameter of <2.5 μm; PM_{10}, fine particulate matter with an aerodynamic diameter of <10 μm; CO, carbon monoxide; SO_2, sulfur dioxide; NO_2, nitrogen dioxide; O_3, ozone; IQR, Interquartile range.

Supplementary Figure 4. Time-lag effects of air pollution on pulse rate

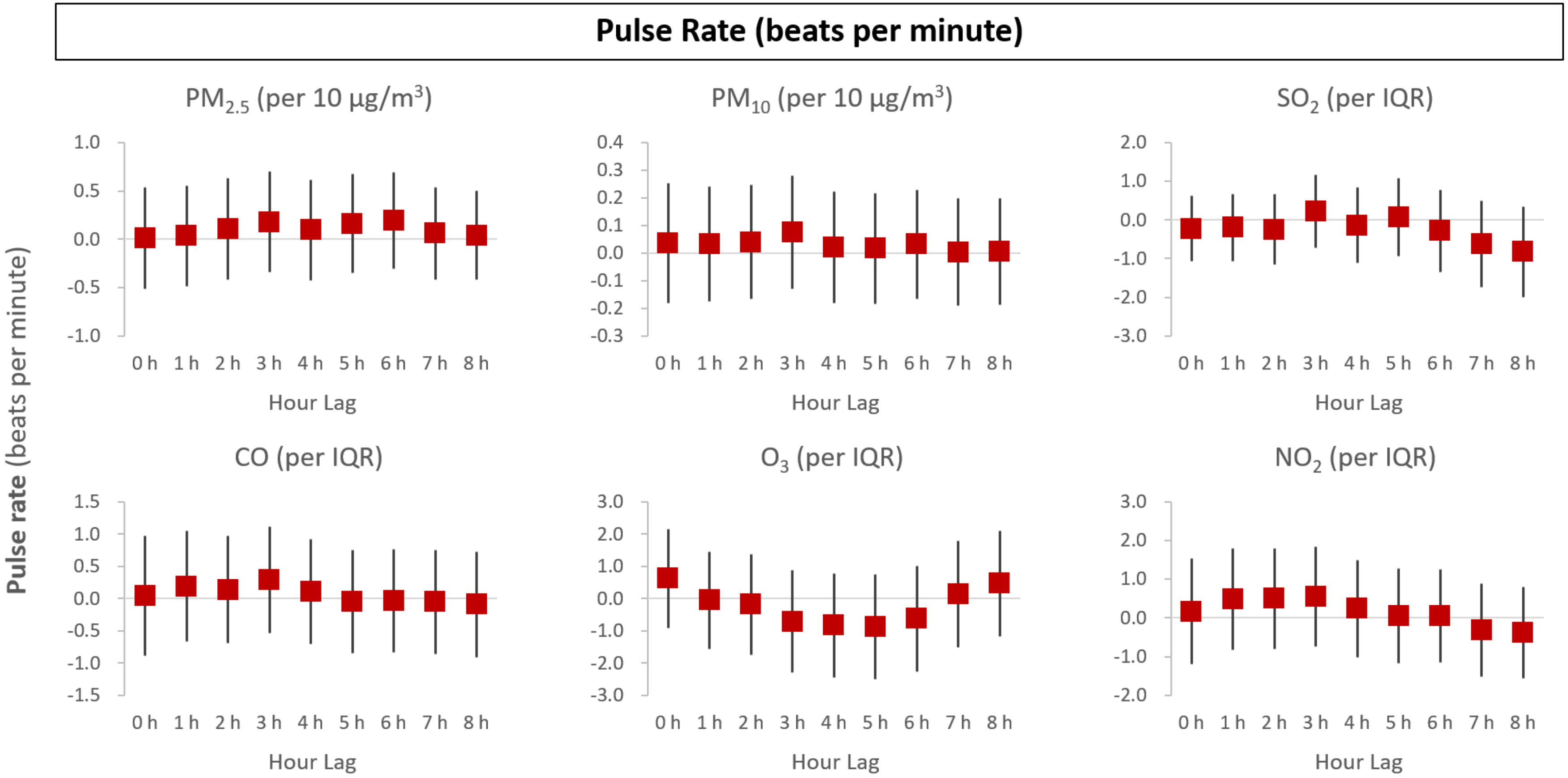

The x-axis represents hour lags, while the y-axis does adjusted effects on systolic blood pressure.

Abbreviations: $PM_{2.5}$, fine particulate matter with an aerodynamic diameter of <2.5 µm; PM_{10}, fine particulate matter with an aerodynamic diameter of <10 µm; CO, carbon monoxide; SO_2, sulfur dioxide; NO_2, nitrogen dioxide; O_3, ozone; IQR, Interquartile range.

Supplementary Figure 5. Subgroup analysis for the associations between air pollutants and systolic blood pressure stratified by body mass index.

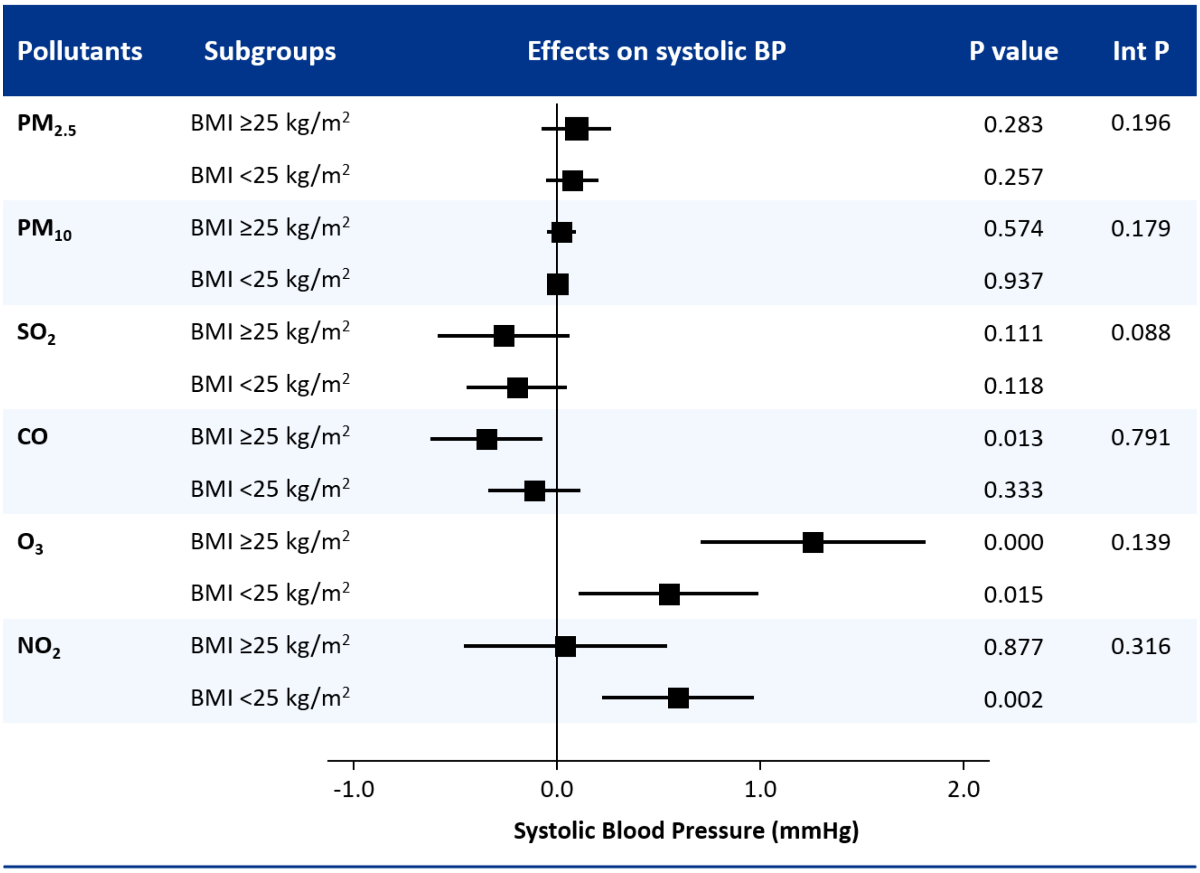

Abbreviations: BMI, body mass index (kg/m^2); Int P, interaction P values; $PM_{2.5}$, fine particulate matter with an aerodynamic diameter of <2.5 µm; PM_{10}; CO, carbon monoxide; SO_2, sulfur dioxide; NO_2, nitrogen dioxide; O_3, ozone.
